# Supplementary material for: Novel Driver Strength Index highlights important cancer genes in TCGA PanCanAtlas patients
Source: PeerJ. 2022 Aug 11;10:e13860. doi: 10.7717/peerj.13860 (PMC9375969; doi:10.7717/peerj.13860)
Supplement: Supplemental Information 2 [file peerj-10-13860-s002.zip › COHORTS/genes plots/age/2021_8_16_14_9_distribution_cohorts_females_75-79.pdf]

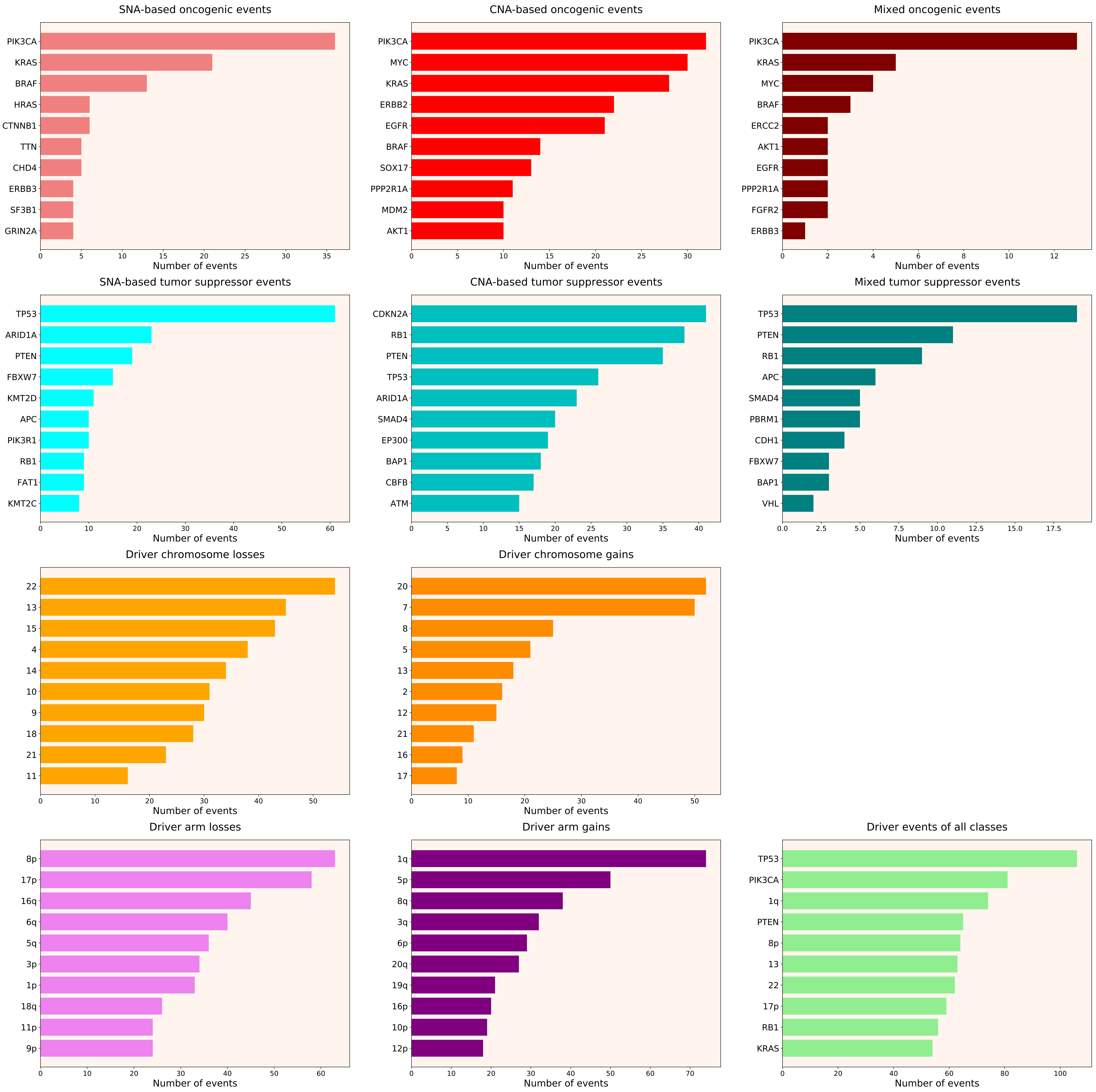
SNA-based oncogenic events
PIK3CA
KRAS
BRAF
HRAS
CTNNB1
TTN
CHD4
ERBB3
SF3B1
GRIN2A
0 5 10 15 20 25 30 35
Number of events
CNA-based oncogenic events
PIK3CA
MYC
KRAS
ERBB2
EGFR
BRAF
SOX17
PPP2R1A
MDM2
AKT1
0 5 10 15 20 25 30
Number of events
Mixed oncogenic events
PIK3CA
KRAS
MYC
BRAF
ERCC2
AKT1
EGFR
PPP2R1A
FGFR2
ERBB3
0 2 4 6 8 10 12
Number of events
SNA-based tumor suppressor events
TP53
ARID1A
PTEN
FBXW7
KMT2D
APC
PIK3R1
RB1
FAT1
KMT2C
0 10 20 30 40 50 60
Number of events
CNA-based tumor suppressor events
CDKN2A
RB1
PTEN
TP53
ARID1A
SMAD4
EP300
BAP1
CBFB
ATM
0 5 10 15 20 25 30 35 40
Number of events
Mixed tumor suppressor events
TP53
PTEN
RB1
APC
SMAD4
PBRM1
CDH1
FBXW7
BAP1
VHL
0.0 2.5 5.0 7.5 10.0 12.5 15.0 17.5
Number of events
Driver chromosome losses
22
13
15
4
14
10
9
18
21
11
0 10 20 30 40 50
Number of events
Driver chromosome gains
20
7
8
5
13
2
12
21
16
17
0 10 20 30 40 50
Number of events
Driver arm losses
8p
17p
16q
6q
5q
3p
1p
18q
11p
9p
0 10 20 30 40 50 60
Number of events
Driver arm gains
1q
5p
8q
3q
6p
20q
19q
16p
10p
12p
0 10 20 30 40 50 60 70
Number of events
Driver events of all classes
TP53
PIK3CA
1q
PTEN
8p
13
22
17p
RB1
KRAS
0 20 40 60 80 100
Number of events
